# Posttraumatic Stress Symptoms and Posttraumatic Growth in Mothers of Children With Intellectual Disability – The Role of Intrusive and Deliberate Ruminations: A Preliminary Report

**DOI:** 10.3389/fpsyg.2019.02011

**Published:** 2019-09-04

**Authors:** Katarzyna Kiełb, Kamilla M. Bargiel-Matusiewicz, Ewa Pisula

**Affiliations:** Faculty of Psychology, University of Warsaw, Warsaw, Poland

**Keywords:** posttraumatic stress symptoms, posttraumatic growth, intellectual disability, mothers, intrusive ruminations, deliberate ruminations

## Abstract

**Background:**

The aim of this study was to find out if mothers of children with intellectual disability (ID) demonstrate symptoms of posttraumatic stress and posttraumatic growth (PTG), and to define the role of intrusive and deliberate ruminations in this area.

**Methods:**

The questionnaire-based study included 85 mothers of children with ID. Three standardized questionnaires were used: Impact of Event Scale-Revised, PTG Inventory, Event-Related Rumination Scale.

**Results:**

Relative to the population norm in Poland, 52% of mothers of children with ID demonstrated at least moderate level of symptoms of posttraumatic stress. The mean level of PTG was high in this group. Intrusive ruminations and mothers’ age served as a predictor for posttraumatic stress symptoms (PTSS). None of the types of rumination correlated with PTG.

**Conclusion:**

Mothers of children with ID demonstrated symptoms of both posttraumatic stress and PTG. Intrusive ruminations proved to be significant predictors for PTSS.

## Introduction

Mothers of children with intellectual disability (ID) experience high levels of parenting stress (Abbeduto et al., 2004; Burke and Hodapp, 2014; Shyam and Govil, 2014) and health problems, including depression and anxiety disorders (Mackey and Goddard, 2006; Gallagher et al., 2008; Miodrag and Hodapp, 2010). The moment of child’s diagnosis of ID is usually associated with powerful negative emotions and tends to be a life-altering occurrence (Hassall et al., 2005). As a result of experienced stress, some mothers may develop posttraumatic stress symptoms (PTSS). PTSS was investigated in parents of children with cancer (Kazak et al., 2004; Phipps et al., 2006), children who experienced serious injury in an accident (Manne, 2009), children with autism (Casey et al., 2012; Roberts et al., 2014) and ID (Hassall et al., 2005). Previous studies show that PTSS observed in parents of children with cancer or medical injury ranges from 11 to 32% (Manne, 2009). Although many of these parents did not meet the full DSM-5 criteria for PTSD, they still experienced severe negative reactions closely associated with a diagnosis of PTSD. These parents reported exposure to a traumatic event and experiencing symptoms from each of the following clusters that are indicative of PTSD: intrusion, hyperarousal and avoidance symptoms (Casey et al., 2012).

There is, however, a lot of variation in how mothers respond to their child’s diagnosis of ID, since parenting tends to also be associated with positive experiences (Lloyd and Hastings, 2009; Arellano et al., 2017). Some researchers have reported posttraumatic growth (PTG) in the mothers (Konrad, 2006; Phelps et al., 2009; Zhang et al., 2013; Counselman-Carpenter, 2017). The founders of the concept of PTG define it as “positive psychological changes experienced as a result of the struggle with traumatic or highly challenging life circumstances” (Tedeschi et al., 2018, p. 3). The model proposed by Tedeschi and Calhoun (1996, 2004), Tedeschi et al. (2018) assumes that PTG involves changes in five areas of individual functioning: personal strength, new possibilities, relationship with others, appreciation of life, spiritual/existential beliefs. The presence of positive changes over time in those areas in mothers of children with autism was also reported by Phelps et al. (2009). They found the most profound changes in positive self-image and psychological wellbeing. Other researchers suggested that PTG in mothers of children with ID primarily involved reappraisal, appreciation of life and interpersonal relationships (Beighton and Wills, 2017; Counselman-Carpenter, 2017).

We still have limited understanding of factors that facilitate PTG in parents of children with ID. The handful of papers on the topic point to the importance of meaning-focused coping (Beighton and Wills, 2017) and social support (Greer et al., 2006). There have also been reports on the importance of problem-focused coping strategy and self-efficacy (Byra et al., 2018).

Individual cognitive processing plays a key role in the development of PTG. The original authors of the construct compare a traumatic event to an “earthquake”: existing cognitive schematic structures are destroyed and need to be rebuilt (Tedeschi and Calhoun, 2004; Tedeschi et al., 2018, p. 4). The change, which is a challenge to the individual, initially triggers intrusive ruminations. These are automatic and involuntary thoughts that appear shortly after trauma and contribute to the emergence of PTSS, such as depressed mood or elevated psychophysiological stimulation (Tedeschi and Calhoun, 2004). As time passes from a traumatic event, the level of distress diminishes and the coping process begins, with intrusive ruminations gradually replaced by deliberate ones. The latter allow for constructive reestablishment of cognitive schematic structures by incorporating into them the traumatic event (Tedeschi et al., 2018). Most authors report positive correlations between intrusive ruminations and the presence of symptoms of posttraumatic stress, and between deliberate ruminations and PTG (Cann et al., 2011; Morris and Shakespeare-Finch, 2011). However, some studies failed to find a relationship between ruminations and PTG (Carboon et al., 2005; Salsman et al., 2009), while others found a negative correlation between intrusive ruminations and PTG (Park et al., 2010).

We are not aware of any analyses of relationships between rumination, PTSS and PTG in mothers of children with ID. Uncovering them would broaden our understanding of the psychological situation of this group of parents and help provide them with support. This study investigated the severity of posttraumatic stress disorder and magnitude of PTG in mothers of children with ID relative to the normal ranges for the Polish population. In addition, based on the theoretical underpinnings of the PTG construct (Tedeschi et al., 2018) and empirical information about other groups, we put forward the following hypotheses on the relationships between ruminations and PTSS and PTG in mothers of children with ID: (1) Intrusive ruminations will predict PTSS severity, and (2) Deliberate ruminations will predict increased PTG.

## Materials and Methods

### Participants

Participants were 86 mothers of children diagnosed with ID. The sample included mothers of children with ID of unknown cause (*n* = 26), Down syndrome (*n* = 16), autism spectrum disorder (*n* = 30) and cerebral palsy (*n* = 14). The age range for mothers was 20–69 years (*M* = 41; SD = 10). The demographics of the sample in the study are shown in Table 1.

**TABLE 1:** Sample characteristics, *N* = 85.

**Variable**	***N* (%)**
***Child diagnosis***	
Autism spectrum disorder	30(35.3%)
Intellectual disability	25(29.4%)
Down syndrome	16(18.8%)
Cerebral palsy	14(16.5%)
***Time since diagnosis***	
6–12 months	8(9.4%)
1–2 years	9(10.6%)
2–5 years	13(15.3%)
>5 years	52(61.2%)
N.D.	3(3.5%)
***Mother’s age***	
20–30 years	8(9.4%)
31–40 years	28(32.9%)
41–50 years	17(20%)
>50 years	10(11.8%)
N.D.	22(25.9%)
***Place of residence***	
City above 500,000 population	17(20%)
City of 101,000–500,000 population	16(18.8%)
City of 51,000–100,000 population	9(10.6%)
City up to 50,000 population	38(44.7%)
N.D.	5(5.9%)
***Mother’s education***	
Primary	4(4.7%)
Vocational	20(23.5%)
Secondary	29(34.2%)
Higher	32(37.6%)
***Mother’s marital status***	
Formal relationship	50(58.8%)
Partnership	13(15.3%)
Single parent	21(24.7%)
N.D.	1(1.2%)

*N.D., no data.*

### Measures

#### Demographics Survey

In the survey, mothers were asked about their age, place of residence, level of education, marital status (single/partnership/formal-married), developmental disorder diagnosed in the child and how long it has been since the diagnosis.

#### Impact of Event Scale

The severity of PTSS was measured using the Impact of Event Scale-Revised (IES-R; Weiss and Marmar, 1997), in Polish adaptation by Juczyński and Ogińska-Bulik (2009). The scale contains 22 statements that assess overall severity of PTSS in three dimensions: Intrusion, Hyperarousal and Avoidance. The presence of symptoms over the last 14 days is assessed on a 5-point Likert scale (from 0 – the symptom has not occurred at all to 4 – the symptom definitely occurred). IES-R has good reliability: Cronbach’s *alpha* is 0.75 for the total scale and satisfactory reliability for subscales: 0.89 for the Intrusion, 0.85 for the Hyperarousal, and 0.78 for the Avoidance. Stability of the IES-R was confirmed by repeated measure after 14 days and it is 0.75, which is satisfactory. Normalization was carried out on a group of 370 who experienced at least one traumatic event: they were firefighters (*n* = 150), women after mastectomy (*n* = 63), men after knee-joint (*n* = 60), parents of children suffering from leukemia (*n* = 67) and prisoners (*n* = 30).

In the assessment of IES-R result, it is advisable to adopt the limit value from which the test result can be considered as a pointing on the occurrence of PTSS. This limit value is 1.5 point (if we calculate the average result) or 33 points (when we calculate the sum for the whole scale). Assuming a limit of 1.5 for the limit value, the result above this point can be treated as a PTSS indicator. With more restrictive approach, which was used in this study, PTSS can be indicated only in person who gets results above 1.5 in each of three dimensions.

#### Posttraumatic Growth Inventory

Posttraumatic growth was measured using a Polish version of the Posttraumatic Growth Inventory (PTGI) by Tedeschi and Calhoun, developed by Ogińska-Bulik and Juczyński (2010). In the instructions, participants were asked to recall the moment of hearing the diagnosis of ID for their child. The inventory consists of 21 items referring to potential positive changes following a traumatic event. The Polish version includes four factors identified through confirmatory factor analysis: changes in self-perception, changes in relationships with others, appreciation of life and spiritual changes (Ogińska-Bulik and Juczyński, 2010). Participants evaluated positive changes in their lives on a scale from 0 – I have not experienced that change to 5 – I have experienced that change to a large degree. Reliability assessed by Cronbach’s *alpha* coefficient is good for the total scale: 0.93, and satisfactory for individual subscales with the following values: 0.87 for changes in self-perception, 0.85 for changes in interpersonal relationships, 0.73 for appreciation of life, and 0.63 for spiritual changes. The normalization covered a total of 730 people aged 16–75 (*M* = 36.5; SD = 14.3). Men accounted for 49.6% and women 50.4%. Altogether the study covered 13 different groups of people who experienced at least one traumatic event, for example: firefighters, police officers and soldiers, people suffering from spinal cord injury and cardiologic conditions, bereaved people, mothers of children suffering with leukemia and mothers of children with Down syndrome.

#### Event-Related Rumination Inventory

Rumination was measured using the Event-Related Rumination Inventory – ERRI (Cann et al., 2011). The Polish version of the inventory was developed by Ogińska-Bulik and Juczyński (2015). ERRI comprises two scales, one that measures intrusive ruminations and one that measures deliberate ones. Participants assessed the presence of ruminations about the moment of their child’s ID diagnosis over the past 14 days on a 4-point Likert scale (0 – not at all, 3 – often). The Polish version of the instrument has good stability: 0.76 and reliability expressed by Cronbach’s *alpha* of 0.96 for the intrusive ruminations subscale and 0.92 for deliberate ruminations subscale. The adaptation and normalization of Polish version of ERRI was attended by a total of 494 people who experienced negative life events. They were oncological patients, parents of oncological children, women-victims of domestic violence, paramedics, and soldiers. The age of the respondents was within 18–67 years (*M* = 38.05; SD = 10.23). Men accounted for 56.5% of respondents and women for 43.5%.

#### Procedure

Recruitment was conducted among mothers of children attending inclusive and special needs educational facilities at the primary school and kindergarten level. Written notes about the study were posted at those facilities. Individuals expressing interest in receiving more detailed information were contacted by email. Participation was anonymous. Mothers were asked to complete a set of questionnaires at home and hand it over in a sealed envelope to the contact person at the facility attended by their child. The recruitment procedure and study protocol were approved by the local Ethics Committee at the Faculty of Psychology, University of Warsaw. The main inclusion criterion was formal diagnosis of mild or moderate ID in the child. The information on the diagnosis and level of ID of children was obtained from preschool or school records with parents’ consent. Out of all collected sets of questionnaires, eight sets were rejected due to having been completed by mothers of children within the intellectual norm but with other disabilities (motor and sensory), and one set completed by a mother of a child with ID was rejected for multiple missing answers. Ultimately, analysis included responses from 85 mothers.

## Results

The distributions of results closely approximated a normal distribution, with the exception of the “intrusive ruminations” variable. The analysis of skewness and kurtosis, however, showed that normal distribution could be assumed for that variable as well (we used limits of skewness and kurtosis ±2). For that reason, correlations between intrusive and deliberate ruminations, and the magnitude of PTSS and PTG were analyzed using Pearson’s *r*. Linear regression analysis was also conducted to find out if ruminations can predict PTSS and PTG. Due to multiple analyses, the significance level for correlations was set at *p* < 0.01. Statistical calculations were done using SPSS.

Descriptive statistics of the magnitude of PTSS, PTG, and ruminations are shown in Table 2. The first step in the analysis was to assess the severity of PTSS in participating mothers. Mean scores of 1.5 points (both for the overall score and for individual subscales of the IES-R scale) was interpreted as cut-off point indicating moderate to high PTSS severity (Juczyński and Ogińska-Bulik, 2009). This severity of PTSS symptoms was found in 52.4% of mothers in the study, compared to low severity in 47.6%.

**TABLE 2:** Descriptive statistics for all key measures, *N* = 85.

**Variable**	***M***	***SD***	***Min.***	***Max.***
PTSS – overall score	37.85	24.81	0	88
Intrusion	14.59	10.28	0	32
Hyperarousal	13.25	9.10	0	32
Avoidance	10.56	6.61	0	24
PTG – overall score	73.36	19.08	33	105
Changes in self-perception	31.9	8.31	1.44	5
Changes in relationships with others	24.32	7.57	0	5
Appreciation of life	12.06	2.74	0	5
Spiritual changes	5.25	3.13	0	5
Intrusive ruminations	16.56	10.68	0	30
Deliberate ruminations	18.68	8.27	0	30

**M*, mean; *SD*, standard deviation; *Min*, minimum score in the sample; *Max*, maximum score in the sample.*

Mean levels of severity of both types of rumination was average, within a sten score of 5 based on the norms for the Polish population (Ogińska-Bulik and Juczyński, 2010).

To determine the level of PTG, participants’ scores were compared with the norms in the IPR scale developed for the Polish population (Ogińska-Bulik and Juczyński, 2010). Mean level of PTG in the study group was within the lower boundary of sten score 7, which is high. The distribution of the overall level of PTG in the study sample was as follows: low in 16 participants (18.8%), moderate in 21 (24.7%), and high in 48 (56.5%).

Table 3 shows the results of analysis of correlations between ruminations and magnitude of PTSS and PTG. Intrusive rumination was significantly correlated positively with the overall level of PTSS and all of its dimensions: intrusion, hyperarousal, and avoidance. There were no correlations between intrusive and deliberate ruminations, and the overall level of PTG or any of its dimensions.

**TABLE 3:** Correlation coefficients between ruminations and PTSS and PTG symptoms, *N* = 85.

**Variables**	**Intrusive ruminations**	**Deliberate ruminations**
PTSS – overall	0.50^∗∗^	0.21
Intrusion	0.53^∗∗^	0.26
Hyperarousal	0.53^∗∗^	0.28
Avoidance	0.34^∗∗^	0.10
PTG – overall	–0.16	0.06
Changes in self-perception	–0.19	0.07
Changes in relationships with others	–0.17	0.09
Appreciation of life	–0.1	0.21
Spiritual changes	–0.15	0.12

*^∗∗^Significant at 0.01 (two-sided).*

The relationships between the overall level of PTSS and overall level of PTG and mothers’ age and time since the child’s diagnosis were also examined (with Spearman Rho coefficient). Correlation analyses yielded no statistically significant relationships between the overall level of PTSS and overall level of PTG. There were statistically significant relationship between PTSS and mothers’ age and time since child’s diagnosis. Results are shown in Table 4.

**TABLE 4:** Correlation coefficients between PTSS, PTG, mother’s age and time since child’s diagnosis, *N* = 85.

	**PTSS**	**PTG**
PTSS	1	–0.04
PTG	–0.04	1
Mothers’ age	–0.44^∗∗^	0.12
Time since child’s diagnosis	−0.26^∗^	0.16

*^∗∗^Significant at 0.01 (two-sided). ^∗^Significant at 0.05 (two-sided).*

To find out which variables can be the predictors of PTSS, stepwise regression was conducted. In the first step, mothers’ age was included and in the second step, intrusive ruminations were added. Both mothers’ age and intrusive ruminations served as the predictors for PTSS (*R* = 0.60; *R*^2^ = 0.36). Results are shown in Table 5.

**TABLE 5:** The results of regression analyses of mothers’ PTSS, N = 85.

**Level of PTSS**							
**Step**	**Predictors**	**B**	**SE**	**β**	***t***	***R***	***R*^2^**
1	Mothers’ age	–1.22	0.31	–0.47	–3.94^∗∗^	0.47	0.22
2	Mothers’ age	–0.9	–0.29	–0.35	−3.02^∗^	0.60	0.36
	Intrusive ruminations	0.88	0.26	0.39	3.38^∗∗^		

*B, unstandardized beta; SE, standard error for the unstandardized beta; β, standardized beta; *t*, *t*-statistics; *R*, coefficient of correlation, *R*^2^, coefficient of determination. ^∗^*p* ≤ 0.01, ^∗∗^*p* ≤ 0.001.*

To check whether the demographic variables have an impact on PTG rates, *t*-Students’ tests were performed. Mothers were divided into two groups: younger (20–35 years) and over 35 years old. Sixty participants provided the information about their age. The age of respondents does not affect the level of PTG: *t*(60) = 0.89, n.i. Younger mothers do not differ in level of PTG (*M* = 68, SD = 23) from older mothers (*M* = 73; SD = 20). Other demographic variables place of residence, level of education and marital status did not differentiate the level of PTG either.

## Discussion

Mothers of children with ID in the study exhibited both the PTSS and the signs of PTG. We also found statistically significant correlations between intrusive ruminations and PTSS but no correlation between ruminations and PTG.

The severity of PTSS was moderate to high in 52.4% of mothers in the sample. This means that over half of the participants were experiencing, on a daily basis, such symptoms as: recurrent images and thoughts related to the negative event (the child being diagnosed with ID), excessive arousal and anxiety, alertness and concentration problems. This is consistent with the results of studies conducted in other countries, which also found elevated levels of PTSS in parents of children with developmental difficulties (e.g., Casey et al., 2012; Roberts et al., 2014). It should be noted, however, that over 40% of mothers experienced those problems with a low severity, which confirms that mothers of children with ID may experience both negative and positive changes after the diagnosis of their children.

The mean overall level of PTG was high, falling within sten score 7. This finding supports the idea that mothers of children with developmental problems have positive experiences related to parenthood (Lloyd and Hastings, 2009; Arellano et al., 2017). The fact that all participants were female may have contributed to such a high level of PTG. According to a meta-analysis by Vishnevsky et al. (2010), sex is significant for the level of PTG: Women typically experience more positive changes following a traumatic event than men.

Mothers’ age and intrusive ruminations predicted PTSS. Since they accounted for of 36% variance in PTSS severity scores, we can conclude that these factors may play an important role in the development of PTSS in mothers of children with ID but there are also many others variables, not included in this study, that can play a crucial role in the adaptation process after potentially traumatic event. This result is consistent with the findings of studies on the relationship between rumination and PTSS in other groups (Cann et al., 2011; Ehring and Ehlers, 2014). This might point to the universal character of correlations between intrusive ruminations and the presence of PTSS, which is only partially associated with the type of traumatic event. Hypothesis 2, which assumed that deliberate ruminations would be predictive of PTG, was not confirmed in our study. The correlation between intrusive rumination and PTG was not statistically significant. The absence of those relationships may be related to the nature of stress experienced by the group in the study. Although the diagnosis of ID in the child can itself be considered traumatic, the stress experienced by parents in the course of caring for their child with ID is of a chronic character, and its level may become elevated in relation to every new challenge parents must face (Duarte et al., 2005; Beighton and Wills, 2017). Thinking back to the difficult moment of receiving the diagnosis and the related feelings and emotions may be an additional burden and result in greater severity of PTSS. If this is indeed the case, cognitive reappraisal of a traumatic event by the mother and deliberate ruminations may not be important for PTG. As suggested by earlier research, social support received by parents and their coping strategies may be more important for the development of PTG (Beighton and Wills, 2017). The role of rumination for PTG is unclear also in the context of other empirical findings. Carboon et al. (2005) and Salsman et al. (2009) also found no correlation between those variables. As noted by Tedeschi et al. (2018), the role of rumination in the process of an individual’s adjustment depends on the type of traumatic event and its subjective cognitive appraisal by that individual. There was no control for the mother’s cognitive appraisal of the child being diagnosed with ID in our study, which prevents us from engaging in a more advanced interpretation of our findings.

The study yielded no statistically significant relationships between the overall level of PTSS and overall level of PTG. This result is consistent with the findings of meta-analysis which showed various relationships between these variables and that these relationships differed according to trauma type and age (Shakespeare-Finch and Lurie-Beck, 2014). The study had several limitations. The sample was small and highly heterogeneous in terms of the mothers’ age and children’s characteristics, including the type of developmental disorder. There is a lack of some demographic information such as number of children in families. As a result, we cannot exclude the influence of this variable on the level of PTG in mothers. It is possible that if they have other children without disabilities, they are an important source of positive experiences of parenting. If mothers have other children without any disabilities, those children may be the source of positive parenting experiences.

As there was no control group, there is little possibility of making inferences about the specifics of the relationships between rumination and PTSS and PTG identified in the study. Future studies would benefit from analyzing the relationships between the age of the child with ID and the presence of PTSS and PTG in the mother, as well as both types of rumination. New insights on the relationships examined here could come from studying fathers and determining the role of parent’s gender on PTSS and PTG and how they relate to rumination. Much more robust data could be collected in longitudinal studies that make it possible to track the dynamics of those relationships over time.

Despite these limitations, the study brings new information that may be useful in the discussion of the consequences of mothers’ experience of trauma associated with their children’s ID. The fact that both the symptoms of PTSS and signs of PTG were found in mothers reflects the complexity of their psychological situation which involves both features indicative of problems with adjustment to parenting a child with ID, and positive experiences related to being a parent. The ambiguous role of rumination, i.e., its involvement in PTSS and lack of significance with respect to PTG, on the other hand, reinforces the view that the relationship between cognitive factors and wellbeing following a trauma is highly complex. The findings presented here may inspire further search for effective ways of supporting families of children with ID, including studies of the significance of trauma associated with the child’s diagnosis of ID for the process of parental adjustment.

## Data Availability

The datasets generated for this study are available on request to the corresponding author.

## Ethics Statement

This study was carried out in accordance with the recommendations of the local Research Ethics Committee at the Faculty of Psychology, University of Warsaw. The protocol was approved by the above mentioned committee. All procedures performed in this study were in accordance with the ethical standards of the institutional research committee and with the Helsinki declaration. Written informed consent was obtained from the participants of this study.

## Author Contributions

KK and KB-M conceived and prepared the study. KK carried out the study. All authors prepared the Introduction section, analyzed the results, and interpreted the results in Discussion section.

## Conflict of Interest Statement

The authors declare that the research was conducted in the absence of any commercial or financial relationships that could be construed as a potential conflict of interest.
